# Mental health effects of caregiving for older parents in the United Kingdom: the roles of social support, neighborhood cohesion, and neighborhood deprivation

**DOI:** 10.1093/geronb/gbag078

**Published:** 2026-05-07

**Authors:** Pilar Zueras, Emily Grundy

**Affiliations:** Centre d’Estudi Demogràfics (CED-CERCA), Universitat Autònoma de Barcelona, Barcelona, Spain; Institute for Social and Economic Research, University of Essex, Colchester, United Kingdom; Institute for Social and Economic Research, University of Essex, Colchester, United Kingdom; (Social Sciences Section)

**Keywords:** Longitudinal, Social strain, Emotional support, Mediation

## Abstract

**Objectives:**

We investigate whether changes in the mental health of adult children providing care for an older parent or parent-in-law are mediated by perceived social support from relatives and friends and perceived neighborhood social cohesion. We also investigate whether neighborhood deprivation modifies the effects of neighborhood social cohesion or independently influences mental health.

**Methods:**

We fit dynamic panel models to analyze changes in the mental health of caregivers using data from 4 annual waves of a nationally representative UK longitudinal study. We distinguish between intensive caregivers providing 20 or more hours of care per week and those providing fewer hours. We controlled for sociodemographic factors associated with mental health and with caregiving. In some analyses, we distinguished between positive aspects of perceived social support and negative effects of social strain.

**Results:**

Higher perceived neighborhood social cohesion was associated with improved mental health for caregivers providing lesser amounts of care and less mental health deterioration for intensive caregivers, irrespective of neighborhood deprivation. Although less intensive caregiving had no direct effect on caregivers’ mental health, their mental health improved because of the association between less intensive caregiving and perceptions of increased support from family, friends, and the neighborhood. However, intensive caregivers reported increased social strain, exacerbating the negative effects of caregiving on mental health.

**Discussion:**

The mental health impacts of caregiving vary by the extent of perceived neighborhood social cohesion and support from family and friends. Stresses and supports of less and more intensive caregivers need separate attention.

Informal care is often crucial for the well-being of older individuals with assistance needs. However, caregiving for older parents or parents-in-law may be emotionally and physically demanding, especially when the care provided is intensive (Hirst, 2005; Lacey et al., 2024; Lee & Gramotnev, 2007; Uccheddu et al., 2019). Although caregiving may nevertheless be a positive experience (Roth et al., 2015), numerous studies suggest that intensive caregiving is associated with deterioration in caregivers’ mental health (Bom et al., 2019; Brenna & Di Novi, 2016; Kaschowitz & Brandt, 2017; Kolodziej et al., 2022; Pinquart & Sorensen, 2006; Schulz & Sherwood, 2008; Stöckel & Bom, 2022).

The impact of caregiving on mental health may be influenced by additional stressors and the availability of supports (Pearlin et al., 1990). Supportive social ties and local environments may enhance caregivers’ coping resources and mediate the impacts of caregiving on perceived stress and mental health. Conversely, strained relationships or poor neighborhood cohesion may exacerbate the impacts of caregiving on stress and deterioration in mental health (Choi & Ailshire, 2024; Wilks & Croom, 2008). Structural factors, such as neighborhood deprivation, may also be important and modify the efficacy of community supports for caregivers (Sui et al., 2022; Zhang et al., 2019).

In this study, we build on previous research on changes in the mental health of adults providing care for a parent or parent-in-law by examining the role of perceived social support from family and friends, also distinguishing perceived positive and negative support, and perceived neighborhood social cohesion. We additionally investigate whether neighborhood deprivation moderates the effects of perceived neighborhood cohesion on caregivers’ mental health. We use data from four rounds of a nationally representative UK longitudinal study, consider intensity (hours) of care provided, and take account of secondary stressors and other relevant socioeconomic and health covariates.

## Family caregiving, the stress process, and social resources

Family caregiving is associated with negative impacts on mental health, including increased depressive symptoms and psychological distress (Cannuscio et al., 2004; Gutiérrez-Sánchez et al., 2023; Pinquart & Sörensen, 2003). However, previous studies have found that caregivers with access to social support have better mental health outcomes than those lacking such support (Cohen & Wills, 1985; del-Pino-Casado et al., 2019; Stafford et al., 2011; Wilks & Croom, 2008).

The stress process model of family caregiving (Pearlin et al., 1990) proposes that caregiving may be a primary source of stress depending on the amount, type, and duration of care provided, as well as characteristics of the caregiver and person cared for, and contextual factors associated with the chance of becoming a caregiver. Secondary stressors, including competing demands, family conflict, or economic problems, may exert additional stress. For example, caregivers facing competing demands from work and family responsibilities have higher risks of deterioration in mental well-being (Bom & Stöckel, 2021; Cannuscio et al., 2004; Kaschowitz & Brandt, 2017; Zueras & Grundy, 2024). As women usually undertake more household and family-related tasks than men, this may mean that female caregivers are at greater risk of deterioration in mental health (Bom et al., 2019; Bom & Stöckel, 2021; Brenna & Di Novi, 2016; Heger, 2017; Hirst, 2005; Pinquart & Sorensen, 2006).

Social networks provide access to forms of social support—instrumental, informational, and emotional (Tajvar et al., 2018; Thoits, 2011) but may also be a source of social strain. Social support may have direct positive effects on well-being and influence mental health through mediation and/or moderation of stress effects. Cohen and Wills (1985), for example, found evidence that esteem, information, or companionship support mediated the appraisal of stressors, which reduced their negative impact on well-being. Thoits (1985) elaborated on this model by reconceptualizing social support as coping assistance acting as a mediator between a challenging event or circumstance and the stress response. Subsequent studies have suggested that social support may also moderate the negative effects of stress on well-being (Amin et al., 2025).

Prior research indicates that social strain, characterized by conflict, frequent criticism, and excessive demands, represents a distinct construct from social support with independent negative impacts (Jackson, 1992). For example, a study of mid-life and older adults in England found that negative exchanges with partners and children were associated with depression, with an impact of a similar magnitude to the beneficial effects of positive exchanges (Stafford et al., 2011).

### Neighborhood influences

Neighborhood factors, such as level of deprivation, are associated with mental health and may influence both the availability and perception of support. Objective neighborhood deprivation is associated with lower perceived social cohesion and poorer mental health (Godhwani et al., 2019; Salvatore & Grundy, 2021; Sui et al., 2022); however, subjective perceptions—such as feeling supported or embedded in the community—frequently show stronger associations with well-being (Yakubovich et al., 2020; Zhang et al., 2019). Living in a deprived neighborhood may heighten exposure to stressors and weaken access to buffering resources, intensifying the impact of stressful events (Cutrona et al., 2006). Conversely, higher perceived neighborhood cohesion is linked to better psychosocial well-being (Kim et al., 2020). Individual characteristics may moderate these associations, and perceptions of cohesion, identification, and neighborhood experiences may mediate them (Hill & Maimon, 2013). Some studies find that emotional support buffers the effects of neighborhood disadvantage in a graduated way (Hill & Maimon, 2013; Schieman & Meersman, 2004), although others suggest that this effect operates primarily in less deprived areas (Elliott, 2000).

Despite substantial research on neighborhood influences on health, fewer studies examine how these factors influence caregivers’ mental health or whether effects vary by caregiving intensity. An exception is a recent study, which found that greater neighborhood cohesion was associated with fewer depressive symptoms among caregivers of people with dementia (Meyer et al., 2022).

### Objectives and hypotheses

In this study, we investigate whether perceived social support from family and friends and neighborhood social cohesion constitute mechanisms linking caregiving for a parent or parent-in-law to changes in mental health. Additionally, we examine the differing effects of perceived emotional support and social strain. We further consider whether neighborhood deprivation modifies the effects of perceived neighborhood social cohesion.

Hypothesis 1 (H1): Perceptions of overall social support may provide caregivers with a sense of validation and recognition and will mediate the relationship between intensive caregiving and changes in caregivers’ mental health (caregiving → overall social support → mental health).

Hypothesis 2 (H2): Perceptions of emotional support and social strain will have independent effects on caregivers’ mental health: intensive caregiving is expected to impact mental health by simultaneously reducing positive emotional resources and increasing interpersonal friction (caregiving → emotional support | social strain → mental health).

Hypothesis 3 (H3): Perceptions of neighborhood social cohesion will mediate the relationship between intensive caregiving and caregivers’ mental health (caregiving → neighborhood social cohesion → mental health).

Hypothesis 4 (H4): The relationship between neighborhood social cohesion and mental health may be moderated by neighborhood deprivation, such that higher levels of perceived social cohesion will have a stronger positive effect on mental health for those living in more deprived neighborhoods (neighborhood social cohesion x neighborhood deprivation → mental health).

## Data and methods

We use nationally representative data from waves 4–7 of the UK Household Longitudinal Survey (UKHLS) to investigate changes in mental health associated with caregiving, and the intensity of caregiving, for older parents or parents-in-law, considering known stressors and other factors that may shape these associations. Social support and neighborhood measures were not simultaneously present across all waves, and for these variables, we used information collected in waves 5 and 6. Information on perceived social support and neighborhood social cohesion was also collected in 2019–21 and 2020–22, but is not used here as the concurrent COVID-19 pandemic may have influenced both caregiving and caregiver mental health, which also resulted in differences in data collection modes.

The selected sample comprised 7,955 adults aged 40–65 who participated in wave 4 of the study—fielded between January 2012 and June 2014—who were also interviewed in waves 5 (2013–15), 6 (2014–16), and 7 (2015–17). Additional inclusion criteria were that respondents had complete information on mental health measures, had a living parent or parent-in-law over the period, and had lived continuously at the same address since wave 5—when social support and neighborhood information was collected—for whom it was possible to link information on neighborhood deprivation.

### Measures

#### Mental health

The main outcome variable was the change in the Mental Component Summary Score of the 12-item Short-Form Health Survey (MCS-12). This validated measure scores mental health from lower to better on a scale of 0 to 100 (Ware et al., 1996).

#### Caregiving

We considered care given to any parent or parent-in-law and created a categorical variable based on reported weekly hours of care provided, distinguishing those not providing care, those providing <20 hours of care per week, and those providing 20+ hours of care per week. For ease of reading, we refer to these categories of lower-hour and higher-hour caregiving as “light/er” or “less intensive” care and “more intensive” or “intensive care,” although we recognize that time alone does not fully reflect the intensity of the caregiver role.

#### Social support

Social support at the individual level was assessed using a combination of variables collected in wave 5, which capture both potential availability of support and perceptions of emotional support and social strain in relationships with relatives and friends. Participants were asked whether they had a partner, friends, and any “immediate family,” “for example, any children, brothers or sisters, parents, cousins, aunts, uncles, and grandparents or grandchildren.” Those who reported having such relationships were asked six questions about how much support or negative exchange they received from them, each with four possible answers (ranging from 1 “not at all” to 4 “a lot”). The specific questions were “How much do they… really understand the way you feel about things?, let you down when you are counting on them?, criticize you?, get on your nerves?” and “How much can you… rely on them if you have a serious problem?, open up to them if you need to talk about your worries?” These questions were asked for each category of connection, so although those on support from a partner relate to a specific individual, those on support from friends or relatives relate to all friends/all relatives. Respondents reporting no relationships were coded as missing (n = 23) and retained to be included in structural equation models computed with full information maximum likelihood estimates (see analytical strategy below).

We derived three measures to estimate the role of perceived social support across all relationship types. First, we computed an averaged measure of all 18 items to estimate the level of overall social support, integrating perceptions of high supportive and low aversive aspects of interactions with family and friends. Next, we created two separate measures distinguishing emotional support from social strain based on perceived positive and negative exchanges (Rouxel et al., 2022). To differentiate the effect of low or high support or strain from the lack of such relationships, particularly in the case of partners, we adjusted the mediation equations for the availability of a coresident partner. The scores of all three scales ranged from 1 to 4 and had good internal consistency (Cronbach’s alpha value for overall social support was 0.80, 0.79 for emotional support, and 0.77 for social strain).

#### Neighborhood social support

Measures of neighborhood perceived social cohesion have been collected triennially in the UKHLS and include two indicators. In the main analysis, we used the Buckner’s Neighborhood Cohesion Instrument (BNCI), which has a Cronbach’s alpha of 0.88, ranges from 1 “lowest cohesion” to 5 “highest cohesion” and draws on the following statements: “I feel like I belong to this neighborhood; if I needed advice about something I could go to someone in my neighborhood; I borrow things and exchange favors with my neighbors; I would be willing to work together with others on something to improve my neighborhood; I plan to remain a resident of this neighborhood for several years; I think of myself as similar to the people that live in this neighborhood; I regularly stop and talk with people in my neighborhood.” Information on BNCI was missing for 13 respondents.

In the sensitivity analysis, we used an alternative measure, the index of Perceived Neighborhood Social Cohesion (PNSC). The PNSC had a Cronbach’s alpha of 0.80, ranging from 4 “lowest social cohesion” to 20 “highest social cohesion” and collected degree of agreement with the following statements: “This is a close-knit neighborhood; people around here are willing to help their neighbors; people in this neighborhood can be trusted; people in this neighborhood generally don’t get along with each other.” Information on PNSC was missing for 62 respondents.

All measures were constructed so that higher scores indicated higher levels of social support, strain, or cohesion.

#### Neighborhood deprivation

We measured neighborhood deprivation using deciles of the official Indexes of Multiple Deprivation (IMD) for small areas (where 1 is the most deprived decile). IMD was anchored to the data collection in wave 6, calculated for 2014 in Wales, 2015 in England, 2016 in Scotland, and 2017 in Northern Ireland, and was provided by each country’s government statistical office. The IMD is a multidimensional local-area indicator based on 38 indicators for seven domains of deprivation relating to income, employment, education and skills, health, crime, housing and services, and living environment, and has been used in recent analyses of perceived neighborhood cohesion and mental health (Salvatore & Grundy, 2021).

#### Control variables

We included time-varying covariates that previous studies have indicated may be associated with stress and mental health, these were: age; long-standing illness or impairment; quintile of household-equivalized income; whether living with children under the age of 16, and employment status. For these variables, the proportion with missing information was less than 1.1%.

### Analytical strategy

**Figure 1:**
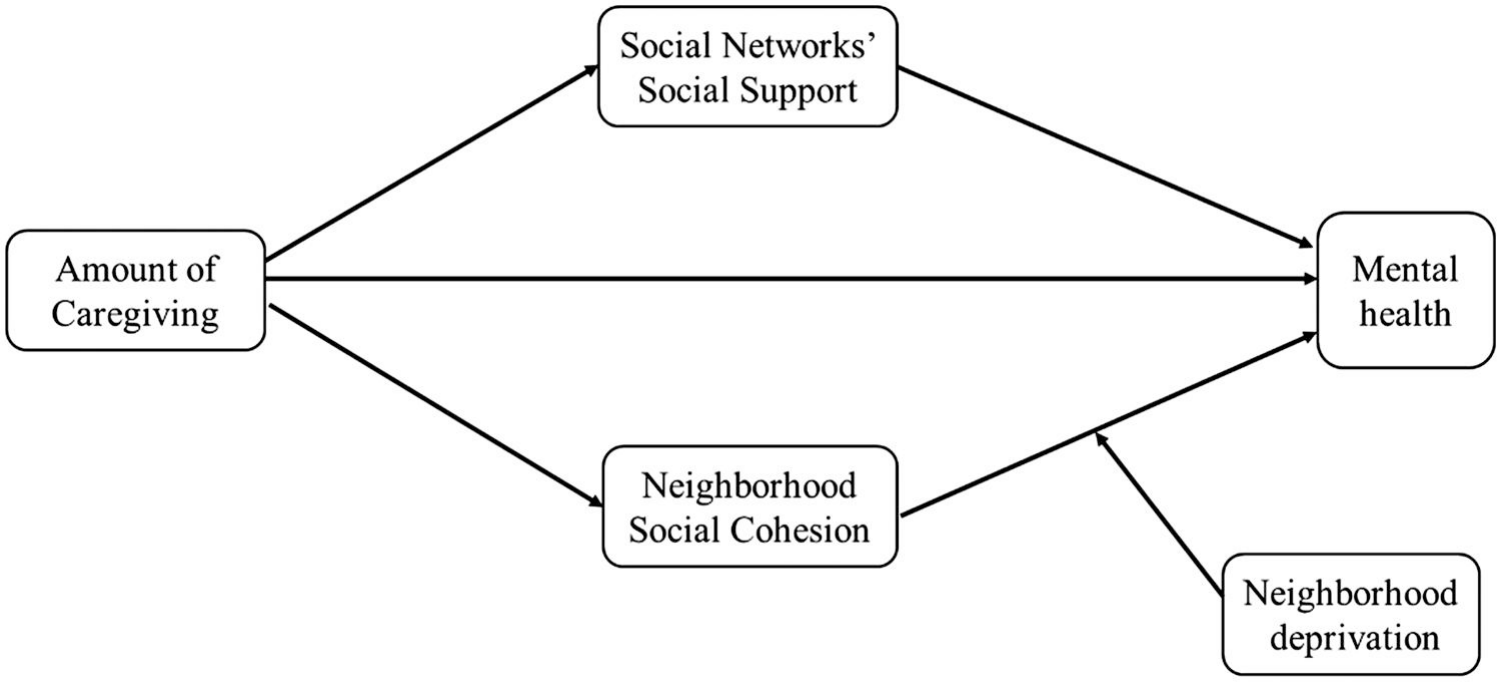
Conceptualized role of individual social support and neighborhood social cohesion in mediating and moderating the association between caregiving and changes in mental health.

We fit dynamic panel models in the structural equation framework to estimate fixed effects of changes in mental health and test for mediating and moderating associations. First, we modeled the effects of known primary stressors (lagged care intensity) on change in mental health, adjusted by covariates. Second, we included measures of perceived support from family and friends and neighborhood social cohesion to test their role in the association between caregiving and mental health. Third, we included the objective measure of the neighborhood context to test whether the effect of perceived neighborhood social cohesion differed by neighborhood deprivation. Finally, we replicated the mediation models using two measures differentiating emotional support and social strain to examine their role as mediators in the relationship between caregiving and mental health. Figure 1 illustrates the conceptual relationships examined in the analytical models.

Analyses were conducted in Stata^®^ version 17.0 (StataCorp, College, Texas) with the xtdpdml command using full information maximum likelihood (FIML) to deal with missing data (Williams et al., 2018).

## Results

### Descriptive results

Table 1 shows characteristics of the analysis cohort by survey wave. The mean age of the sample at baseline was 50 years, and 55.2% were women. The proportions engaged in parental caregiving varied across waves, ranging from 14.8% to 17.2% providing less intensive care, and from 1.8% to 2.0% providing more intensive care (20 or more hours of care per week). During the observation window, 35.6% of women provided lighter care at some point compared with 25.3% of men, and 6.7% of women and 2.5% of men provided more intensive care. Mean scores for overall social support and positive emotional support were higher than scores for negative perceptions of social strain. The mean values for perceived neighborhood cohesion were also high, with the BNCI ranging from 1 to 5, with a mean value of 3.70, and scores on the PNSC scale ranging from 4 to 20, with an observed mean of 14.95. Most study members lived in less deprived neighborhoods, with a mean IMD decile score of 6.10; however, average household incomes were relatively low. Most people lived with a partner, and more than 30% lived with children under the age of 16. Approximately one-third of the study population had a long-standing illness at some point during the observation period, and over 75% were employed.

**Table 1:** Characteristics of the analytical sample by study wave (*N* = 7,955).

Variable	Wave 4	Wave 5	Wave 6	Wave 7
**Women, *N* (%)**	4,391 (55.20)			
**Age**	49.98 (6.77)			
**Mental component summary**	49.66 (9.39)	49.36 (9.50)	50.05 (9.44)	49.55 (9.68)
**Caregiver status, *N* (%)**				
Non-caregivers	6,583 (82.75)	6,570 (82.59)	6,450 (81.08)	6,639 (83.46)
Less intensive caregivers	1,214 (15.26)	1,243 (15.63)	1,366 (17.17)	1,175 (14.77)
More intensive caregivers	158 (1.99)	142 (1.79)	139 (1.75)	141 (1.77)
**Overall social support**		3.22 (0.40)		
**Emotional support**		3.16 (0.54)		
**Social strain**		1.72 (0.45)		
**Buckner’s neighborhood cohesion instrument (BNCI)**			3.70 (0.67)	
**Neighborhood social cohesion (PNSC)**			14.95 (2.52)	
**Index of multiple deprivation decile (1 = most deprived), circa 2015**		6.1 (2.76)		
**Household net-income quintile equivalized (1 = highest 5 = lowest)**	3.41 (1.37)	3.4 (1.38)	3.46 (1.33)	3.44 (1.36)
**Living with a partner, *N* (%)**	6,355 (79.89)	6,353 (79.86)	6,336 (79.65)	6,337 (79.66)
**Living with children under the age of 16, *N* (%)**	2,880 (36.20)	2,652 (33.34)	2,400 (30.17)	2,186 (27.48)
**Long-standing illness or impairment, *N* (%)**	2,699 (33.94)	2,632 (33.06)	2,710 (34.22)	2,708 (34.15)
**Employed, *N* (%)**	6,272 (78.84)	6,212 (78.09)	6,166 (77.51)	6,043 (75.96)

*Note*. Categorical variables statistics display the number (*N*) and unweighted proportions (%), while the mean and standard deviation (SD) are displayed for continuous variables and those treated as quasi-continuous in the analysis.

### Model results

#### Preliminary analysis

Preliminary bivariate analyses were undertaken to inform the specification of mediation and moderation pathways in subsequent structural equation models (SEM), which indicated that mediation pathways were more strongly supported than moderation effects (Supplementary Table 1). In preliminary SEM analyses (Supplementary Table 2), we fit step-by-step models to examine the relationship between caregiving intensity and changes in mental health (Model 1), along with the mediating roles of social support and neighborhood cohesion variables tested one at a time (Models 2–4), and the potential moderating role of neighborhood deprivation (Model 5) including the interaction between neighborhood cohesion and neighborhood deprivation. All models were adjusted for age, long-standing illness, and household income. Full models were estimated separately for overall social support (Model 6) and for emotional support and social strain as distinct constructs (Model 7). Model 8 incorporated additional adjustments to allow for possible effects of competing demands on caregivers (parenting and employment), which were not significant. Results from these models are shown in Supplementary Table 2.

#### Results from selected models

**Figure 2:**
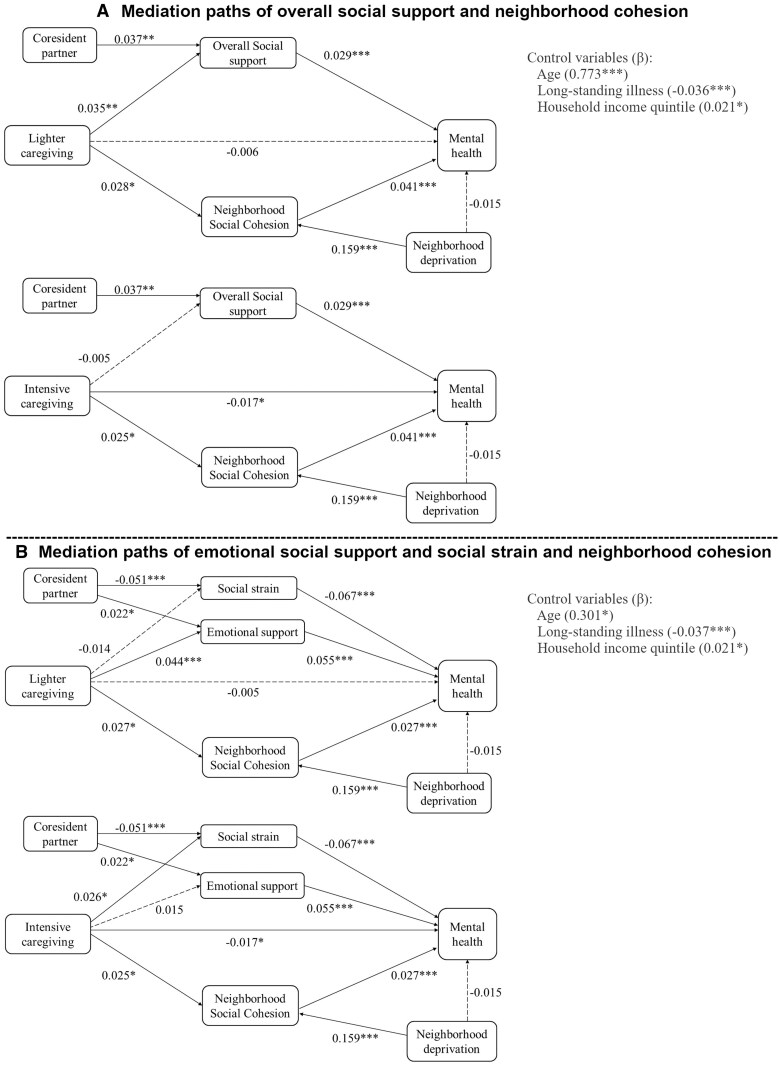
Direct and indirect effects of caregiving, social support, and contextual factors on changes in mental health by intensity of care and different indicators of social support from relatives and friends. (A) Mediations paths of overall social support and neighborhood cohesion. (B) Mediations paths of emotional social support and social strain and neighborhood cohesion. Numerical values represent standardized coefficients (β). Solid lines indicate significant associations; dashed lines indicate non-significant associations. *p < .05. **p < .01. ***p < .001.

Figure 2 presents path diagrams with results from Models 6 and 7 showing direct and indirect effects of caregiving; perceived support from family/friends (adjusted for the presence of a coresident partner); perceived neighborhood social cohesion; and neighborhood deprivation, on changes in mental health, distinguishing less and more intensive caregivers. The models also control for age, long-standing illness, and household income quintile. We present standardized coefficients (β*) as these facilitate comparison of effects when continuous variables are measured in different units (Supplementary Table 3).

Figure 2A shows that more overall social support was positively associated with better mental health as indicated by a higher MCS score (β* = 0.035, *p *< .01). Having a partner had a positive influence on social support (and so indirectly on mental health). Less intensive caregiving was positively associated with overall perceived social support, but the association between more intensive caregiving and social support was not significant. Both lighter and more intensive caregiving were positively associated with perceived neighborhood cohesion, which was associated with improvements in mental health. The direct effect of caregiving on mental health was negative for more intensive caregivers but not significant for lighter caregivers. Perceptions of neighborhood cohesion were higher in less deprived neighborhoods. As a result, there was an indirect association between neighborhood deprivation and change in mental health (mediated by perceived neighborhood cohesion) even though neighborhood deprivation was not directly associated with changes in caregiver mental health.

Figure 2B presents the results from Model 7, which distinguishes positive and negative dimensions of exchanges with the individual’s social network. These results indicate that lighter caregiving increased perceptions of emotional support (β* = 0.044, *p *< .001), which improved mental health, whereas intensive caregiving increased perceptions of social strain (β* = 0.026, *p *< .05), which had a detrimental effect on mental health (β* = −0.67, *p *< .001). Living with a partner was associated with increased perceptions of emotional support and was protective against perceptions of social strain. Results also show that perceived neighborhood social cohesion consistently mediated the effects of both lighter (β* = 0.027, *p *< .05) and more intensive caregiving (β* = 0.025, *p *< .05) on change in mental health, as well as the effects of neighborhood deprivation (β* = 0.016, *p *< .001). However, the direct effect of neighborhood cohesion on MCS was smaller (β* = 0.027, *p *< .01) than that of emotional support (β* = 0.055, *p *<.001) or social strain (β* = −0.067, *p *< .001).

Results of mediation tests using the Baron and Kenny (1986) and the Zhao et al. (2010) approach are presented in Supplementary Table 4. According to the mediation test in Zhao et al. (2010), about 19% of the effect of lighter caregiving on change in MCS was mediated by overall social support (Model 6), about 53% was mediated by emotional support, and 13% by perceived neighborhood cohesion (Model 7). Only about 9% of the effect of intensive caregiving on change in MCS was mediated by the damaging effects of social strain. Perceived neighborhood cohesion was protective and partially offset the direct detrimental effects of intensive care on mental health, although this mediation effect was relatively small (5%).

These results provide support for some but not all of our hypotheses. H1 was not confirmed, as overall perceived social support did not mediate the relationship between intensive caregiving and caregivers’ mental health, although it did mediate the association between lighter caregiving and mental health. Confirming H2, the role of perceptions of positive and negative exchanges of social support differed by the intensity of caregiving. Compared to non-caregivers, lighter caregivers experienced an improvement in mental health mediated by indirect effects of perceived increased emotional support from relatives and friends. In contrast, intensive caregivers experienced a deterioration in mental health, and also indirectly by increased perceptions of social strain in relationships with relatives and friends. H3 was also confirmed, with results indicating that perceived neighborhood cohesion mediated positive effects of both intensive and lighter caregiving on mental health. In contrast, H4 was not supported as we found no evidence that neighborhood deprivation modified the association between perceived neighborhood cohesion and mental health. However, the effects of neighborhood deprivation on mental health were mediated by perceived neighborhood cohesion. See Supplementary Figure 1 for a summary of the significant associations and direction of potential mediators related to the study hypotheses.

#### Sensitivity analyses

The small size of the group providing intensive care may reduce the precision of the estimates. However, robustness checks fit for the final model using robust standard errors produced the same significant results, thus supporting the validity of our findings. We conducted several other sensitivity analyses to validate the main findings. First, before the rejection of the hypothesis of a moderating effect of neighborhood deprivation, the IMD was recoded into a three-category variable to capture any potential differences in associations between the most and least deprived neighborhoods, in comparison to the central 50% of neighborhoods in the analytical sample. However, no significant effect of interaction terms was identified (results not shown). Second, we stratified Model 7 by gender (Supplementary Table 3), and differences emerged primarily in the significance of the roles of social support and strain: for women, providing intensive care was associated with increased social strain, while for men, lighter caregiving was associated with greater emotional support. Next, we used the alternative measure of neighborhood social cohesion (PNSC) and confirmed that it had a significant positive direct effect on mental health and mediated the positive effect of living in less deprived neighborhoods, as found using the BNCI in the main analysis. However, the PNSC measure did not mediate any form of caregiving and mental health (Supplementary Table 5). Finally, we conducted sensitivity analyses fitting the final full model to predict responses to the 12-question General Health Questionnaire (GHQ) as an alternative measure of overall psychological well-being (Goldberg et al., 1997). The GHQ has values ranging from 0 (least distressed) to 36 (most distressed), in the opposite direction to MCS (Supplementary Table 6). We found that neither lighter nor more intensive caregiving was significantly associated with changes in GHQ scores. However, the model confirmed all the indirect mediation pathways found for lighter and intensive caregiving, showing increasing perceptions of neighborhood cohesion and emotional support for lighter caregivers, but increasing social strain for intensive caregivers, with equivalent effects on caregivers’ psychological well-being.

## Discussion

This study contributes to the literature on health effects of caregiving by testing well-defined hypotheses—derived from established theoretical and empirical frameworks—regarding the roles of individual social support, neighborhood cohesion, and neighborhood deprivation in mediating or moderating the impact of caregiving for a parent or parent-in-law on changes in mental health. Drawing on nationally representative longitudinal UK data, we differentiated between “lighter” caregiving and “more intensive” caregiving of 20 or more hours per week.

Our findings align with the stress process model (Pearlin et al., 1990) and the hypothesis of the mediating effect of social support (Cohen & Wills, 1985). Intensive caregiving was found to have a direct, detrimental effect on mental health that persisted even after accounting for individual and contextual factors. In contrast, lighter caregiving had no significant direct impact on change in mental health but showed indirect benefits for mental health via enhanced perceptions of social support at both the individual and neighborhood levels.

Mediation analyses indicated that emotional support from friends and relatives, as well as perceived neighborhood social cohesion, was associated beneficially with changes in mental health, while social strain in close relationships had the opposite effect. Lighter caregiving was associated with greater perceived emotional and overall social support, while intensive caregiving was linked to higher social strain, amplifying its negative impact on mental health. Caregiving, regardless of intensity, was positively associated with greater perceived neighborhood cohesion, suggesting that caregivers may become more involved in their local communities and find support that enhances their capacity to cope with the stresses of the caregiving role and so offset potentially harmful effects on their mental health.

Contrary to our expectations, neighborhood deprivation did not moderate the relationship between neighborhood cohesion and mental health. However, in line with Elliot’s (2000) results, we found an indirect effect: individuals in less deprived neighborhoods perceived higher levels of neighborhood cohesion, which in turn was associated with better mental health outcomes.

Our results support previous research indicating that caregiving intensity significantly influences changes in mental health (Kolodziej et al., 2022; Zueras & Grundy, 2024). The buffering role of perceived neighborhood cohesion suggests that community environments may mitigate some of the stress associated with intensive caregiving. The indirect benefits of lighter caregiving may be explained by increased social connectedness and the sense of purpose or validation derived from helping, similar to reported benefits from volunteering (Nichol et al., 2023). Although we could not directly examine specific activities associated with lighter caregiving, such as running errands or escorting a parent to appointments, these tasks may foster interactions that enhance community integration (Elliott, 2000).

Perceived neighborhood cohesion emerged as a particularly important mediator across all analyses. Higher perceived cohesion was consistently associated with better mental health, reinforcing its potential as a target for community-level interventions to support caregivers. These findings are consistent with other research (Hill & Maimon, 2013; Wang et al., 2021). Although we cannot ascertain causal mechanisms in this observational study, the findings point to a protective role of cohesive communities.

While greater perceived emotional and overall social support positively influenced the mental health of lighter caregivers, these supports did not buffer the adverse effects of intensive caregiving. This result diverges from that of a recent review and meta-analysis (Gutiérrez-Sánchez et al., 2023), but that review considered studies on all types of caregivers, not just those for older relatives, and did not distinguish between effects for “lighter” and “more intensive” caregivers. Our results suggest that while emotional support enhances well-being under lower caregiving demand, it may be insufficiently protective when demands are high. Our results align with previous studies (Jackson, 1992; Umberson et al., 1996) in showing that the effects of social relationships on mental health vary depending on whether they are perceived as supportive or stressful. Using three measures of social support—overall social support, emotional support, and social strain—provides a more nuanced understanding of the effect of social relationships on change in caregiver mental health than analyses relying on aggregate perceptions or indicators of support availability, which implicitly assume that interactions with relatives and friends will be supportive. Our study highlights the importance of considering the quality of social interactions, revealing how positive and negative interaction qualities are differentially related to caregiving and mental health.

### Strengths and limitations

Strengths of this study include, first, the use of a large, nationally representative longitudinal dataset with rich information on caregiving, social support, perceptions of neighborhood cohesion, objectively measured neighborhood deprivation, and mental health, enabling a nuanced analysis of both direct and indirect pathways from caregiving to mental health. Second, by disaggregating social support into emotional support and social strain, using validated instruments to measure perceived neighborhood cohesion, and external objective measures of neighborhood deprivation, the study advances understanding of the complex social pathways through which caregiving for parents may influence mental health. The results provide further evidence that the intensity of caregiving influences perceptions of both individual and community social experiences. Third, the gender-stratified models included in sensitivity analyses add depth to the analysis by revealing important gender-specific patterns, suggesting that men may benefit emotionally from providing low-intensity care, whereas women may face heightened stress in relationships under more intensive caregiving demands. Fourth, we undertook sensitivity analyses using different measures of neighborhood cohesion and mental health. Although there were some differences in results using these alternative indicators, the main conclusions were upheld.

However, the study also has limitations. The reliance on self-reported measures introduces the possibility of reporting bias, particularly in subjective assessments of support and neighborhood cohesion. These assessments might be influenced by respondents’ mental health, with the risk of reverse causality: for example, caregivers experiencing depression may perceive their social ties and neighborhood cohesion less favorably. Additionally, the data collected on social support and social strain related to respondents’ overall networks, rather than to specific individuals. Moreover, caregiving was assessed only in terms of intensity as indicated by hours spent caregiving and relationship (parent or parent-in-law), without capturing variations in the quality of relationships, care recipient needs, or duration of caregiving. Also, we recognize that terming 20 hours of care per week “light” or “lighter” care may inadequately capture the extent of caregiver burden, as intensity varies with task type, condition severity, relationship, and whether care is shared. Finally, while longitudinal, the observational design limits inference of causal effects.

## Conclusion

We analyzed associations between caregiving for a parent or parent-in-law and the potential protective role of social support from family, friends, and the neighborhood environments in supporting caregivers’ mental well-being. The findings highlight the importance of distinguishing between caregiving intensities and considering both positive and negative aspects of social exchanges with relatives and friends, as they mediate the effects of caregiving on mental health in opposite directions. Lighter caregiving was associated with improved perceived emotional support, particularly among men, associated with improved mental health, whereas intensive caregiving was associated with increased social strain, especially among women, which was associated with worsened mental health. In contrast, increased perceptions of neighborhood social cohesion positively mediated the effects of both light and intensive caregiving. This suggests that interventions to improve the feelings of psychological sense of community might benefit the mental health of caregivers, although establishing this would require further research designed to test interventions rather than relying on analyses of observational data.

## Supplementary Material

gbag078_Supplementary_Data

## Data Availability

Data are available at the University of Essex, Institute for Social and Economic Research (2024). Understanding Society: Waves 1–14, 2009–2023 and Harmonized BHPS: Waves 1–18, 1991–2009. 19th Edition. UK Data Service. SN: 6614, doi: 10.5255/UKDA-SN-6614-20. The linked information on the small area Indexes of Multiple Deprivation was provided by the UK Data Service under a special license agreement (ref. 221104—Social connectedness and caregiver’s mental health).
