# Supplementary material for: Mental health effects of caregiving for older parents in the United Kingdom: the roles of social support, neighborhood cohesion, and neighborhood deprivation
Source: J Gerontol B Psychol Sci Soc Sci. 2026 May 7;81(7):gbag078. doi: 10.1093/geronb/gbag078 (PMC13248945; doi:10.1093/geronb/gbag078)
Supplement: gbag078_Supplementary_Data [file gbag078_supplementary_data.pdf]

Mental Health Effects of Caregiving for Older Parents in the UK: The Roles of Social Support, Neighborhood Cohesion, and Neighborhood Deprivation

Index

Table 1S. Bivariate associations between caregiving, social support variables, neighborhood factors, and mental health (Mental Component Score - MCS) to test potential mediation and moderation paths.

Panel A. Bivariate associations between independent variables and mental health, and interactions between caregiving intensity and neighborhood factors to test for moderation paths.

Panel B. Interactions between caregiving intensity and perceived social support to test for moderation paths.

Panel C. Complementary associations between caregiving intensity and social support variables, neighborhood factors to test for mediation paths.

Table 2S. Abbreviated results of selected dynamic panel models including mediation equations. Models 1 to 8. Unstandardized adjusted coefficients.

Table 3S. Model 6 and 7 and Model 7 stratified by gender. Abbreviated results with standardized coefficients.

Table 4S. Mediation test using both the Baron and Kenny, and the Zhao, Lynch and Chen approaches.

Table 5S. Abbreviated results of selected dynamic panel models including mediation equations using an alternative measure of neighborhood cohesion (Perceived Neighborhood Social Cohesions, PNSC). Standardized adjusted coefficients.

Table 6S. Abbreviated results of Model 7 fitted to the General Health Questionnaire (GHQ). Standardized adjusted coefficients.

Figure 1S. Path diagrams with the significant associations between caregiving, social support, neighborhood social cohesion, and mental health.

Table 1S. Bivariate associations between caregiving, social support variables, neighborhood factors, and mental health to test potential mediation and moderation paths.

Panel A. Bivariate associations between independent variables and mental health, and interactions between caregiving intensity and neighborhood factors to test for moderation paths.

	Model 1<br>MCS	Model 2<br>MCS	Model 3<br>MCS	Model 4<br>MCS	Model 5<br>MCS	Model 6<br>MCS	Model 7<br>MCS	Model 8<br>MCS	Model 9<br>MCS	Model 10<br>MCS
**Lagged Caregiving**										
Light care	0.478									
	(0.283)									
Intensive care		-4.458***								
		(0.950)								
**Social network perceived support**										
Overall social support			7.044***							
			(0.287)							
Emotional support				4.052***						
				(0.216)						
Social strain					-5.291***					
					(0.258)					
**Neighborhood factors**										
Index of Multiple Deprivation (IMD)						0.375***				
						(0.039)				
Buckner's Neighborhood Cohesion Instrument (BNCI)							2.309***			
							(0.173)			
Interaction: BNCI								2.117***		
								(0.172)		
BNCI # IMD								-0.008		
								(0.065)		
IMD								0.290***		0.269***
								(0.039)		(0.040)
Neighborhood Social Cohesion (PNSC)									0.496***	
									(0.048)	
Interaction: PNSC										0.414***
										(0.050)
PNSC # IMD										-0.021
										(0.017)
N	7955	7955	7933	7933	7932	7955	7942	7942	7893	7893
R-squared	0.000	0.004	0.088	0.053	0.064	0.012	0.027	0.034	0.018	0.024

Panel B. Interactions between caregiving intensity and perceived social support to test for moderation paths.

	Model 11<br>MCS	Model 12<br>MCS	Model 13<br>MCS	Model 14<br>MCS	Model 15<br>MCS	Model 16<br>MCS	Model 17<br>MCS	Model 18<br>MCS	Model 19<br>MCS	Model 20<br>MCS	Model 21<br>MCS	Model 22<br>MCS
Overall social support	7.223***	7.065***	7.112***	6.982***								
	(0.314)	(0.288)	(0.317)	(0.290)								
Lagged light care	0.246				0.268		0.391					
	(0.277)				(0.281)		(0.277)					
Lagged light care #<br>Overall social support	-1.351<br>(0.752)											
Lagged intensive care		-4.305***				-4.679***		-4.088***				
		(0.920)				(0.919)		(0.953)				
Lagged intensive care #<br>Overall social support		-1.101<br>(2.090)										
Light care			0.596*						0.566*		0.742**	
			(0.269)						(0.274)		(0.271)	
Light care #<br>Overall social support			-0.562<br>(0.727)									
Intensive care				-2.834**						-3.164***		-2.760**
				(0.900)						(0.919)		(0.930)
Intensive care #<br>Overall social support				2.079<br>(2.090)								
Emotional support					4.151***	4.042***			4.056***	4.002***		
					(0.236)	(0.216)			(0.239)	(0.218)		
Lagged light care #<br>Emotional support					-0.735<br>(0.578)							
Lagged intensive care #<br>Emotional support						0.914<br>(1.625)						
Social strain							-5.404***	-5.299***			-5.376***	-5.254***
							(0.281)	(0.258)			(0.281)	(0.260)
Lagged light care #<br>Social strain							0.889<br>(0.680)					
Lagged intensive care #<br>Social strain								1.523<br>(2.042)				
Light care #<br>Emotional support									-0.112<br>(0.536)			
Intensive care #<br>Emotional support										2.291<br>(1.585)		
Light care #<br>Social strain											0.635<br>(0.696)	
Intensive care #<br>Social strain												-0.858<br>(1.944)
N	7933	7933	7933	7933	7933	7933	7932	7932	7933	7933	7932	7932
R-squared	0.089	0.092	0.089	0.090	0.054	0.058	0.064	0.067	0.054	0.056	0.065	0.065

Panel C. Complementary associations between caregiving intensity and social support variables, neighborhood factors to test for mediation paths.

	Model 23 Overall social support	Model 24 Emotional support	Model 25 Social strain	Model 26 Buckner's Neighborhood Cohesion Instrument (BNCI)	Model 27 Neighborhood Social Cohesion (PNSC)
**Lagged Caregiving**					
Light care	0.039**	0.063***	-0.018		
	(0.012)	(0.016)	(0.013)		
Intensive care	-0.020	0.055	0.095*		
	(0.040)	(0.049)	(0.044)		
Light care				0.051*	0.029
				(0.020)	(0.075)
Intensive care				0.126*	-0.145
				(0.061)	(0.247)
**Index of Multiple Deprivation**				0.039***	0.213***
				(0.003)	(0.010)
N	7933	7933	7932	7942	7893
R-squared	0.001	0.002	0.001	0.027	0.055

Table 2S. Abbreviated results of selected dynamic panel models including mediation equations. Unstandardized adjusted coefficients.

**MCS**<br>Variable	model 1	model 2	model 3	model 4	model 5	model 6	model 7	model 8
**Direct effects of caregiving**								
Lagged MCS	0.121***	0.120***	0.136***	0.125***	0.123***	0.130***	0.138***	0.137***
**Lagged Caregiving**								
(ref. No caregiving)								
Light care	-0.213	-0.182	-0.196	-0.169	-0.171	-0.165	-0.180	-0.186
Intensive care	-1.175*	-1.138*	-1.121*	-1.143*	-1.142*	-1.175*	-1.156*	-1.148*
Control variables included								
Age		-0.332***	-0.479***	0.037	0.038	1.085***	0.422*	0.440*
Long-standing illness		-0.749***	-0.748***	-0.747***	-0.747***	-0.740***	-0.740***	-0.735***
Equivalized household income quintiles		0.158*	0.152*	0.150*	0.151*	0.148*	0.146*	0.124
Empoyed (vs non-employed)								0.424
Living with a children under the age of 16								-0.560
**Direct effects of mediators**								
**Social network perceived support**								
Overall social support		-0.240***				0.683***		
Emotional support			0.595***				0.993***	0.993***
Social strain			-1.716***				-1.350***	-1.350***
**Neighborhood perceived support**								
Buckner's Neighborhood Cohesion Instrument				0.320***	0.237***	0.573***	0.407***	0.407***
Index of Multiple Deprivation (IMD)				-0.075*	-0.045	-0.051	-0.059	-0.059
Interaction term with IMD					0.003			

**Mediators' equations**								
**Indirect effects of caregiving**								
**Social network perceived support**								
**Overall social support**								
Lagged light care		0.039**				0.039**		
Lagged intensive care		-0.015				-0.014		
Having a coresident partner		0.037***				0.037***		
**Emotional support**								
Lagged light care			0.063***				0.063***	0.063***
Lagged intensive care			0.059				0.059	1.059
Having a coresident partner			0.031*				0.031*	0.031*
**Social strain**								
Lagged light care			-0.017				-0.017	0.983
Lagged intensive care			0.087*				0.087*	0.087*
Having a coresident partner			-0.059***				-0.059***	-0.059***
**Neighborhood perceived support**								
**Buckner's Neighborhood Cohesion Instrumen**								
Lagged light care				0.051*	0.051*	0.051*	0.051*	0.051*
Lagged intensive care				0.126*	0.126*	0.126*	0.126*	0.126*
Index of Multiple Deprivation (IMD)				0.039***	0.039***	0.039***	0.039***	0.039***
**Statistics**								
N	7955	7955	7955	7955	7955	7955	7955	7955
Akaike Information Criterion	205000	371000	385000	409000	454000	425000	439000	439000
Bayesian Information Criterion	205000	372000	387000	411000	456000	426000	441000	441000

Note: Stars indicate statistical significance *p<0.05, **p<0.01, ***p<0.001.

Table 3S. Models 6 and 7, and Model 7 stratified by gender. Abbreviated results with standardized coefficients.

MCS<br>Variable	Model 6	Model 7	Model 7<br>Male	Model 7<br>Female
**Direct effects of caregiving**				
Lagged MCS		0.137	0.136	0.14
**Lagged Caregiving**				
(ref. No caregiving)				
Light care	-0.006	-0.007	-0.001	-0.011
Intensive care	-0.017*	-0.017*	-0.017	-0.018
Control variables	x	x	x	x
**Direct effects of mediators**				
**Social network perceived support**				
Overall Social Support	0.029***			
Emotional support		0.057***	0.069***	0.053***
Social strain		-0.064***	-0.074***	-0.060***
**Neighborhood perceived support**				
Buckner's Neighborhood Cohesion Instrument	0.041***	0.029***	0.027***	0.035***
Index of Multiple Deprivation (IMD)	-0.015	-0.017	-0.001	-0.031
**Mediators' equations**				
**Indirect effects of caregiving**				
**Social network perceived support**				
**Overall social support**				
Lagged light care	0.035**			
Lagged intensive care	-0.005			
Having a coresident partner	0.037**			
**Emotional support**				
Lagged light care		0.042*	0.033*	0.020
Lagged intensive care		0.015	-0.028	-0.007
Having a coresident partner		0.023*	0.068***	0.014
**Social strain**				
Lagged light care		-0.014	-0.022	-0.022
Lagged intensive care		0.027*	0.015	0.033*
Having a coresident partner		-0.052***	-0.049**	-0.053**
**Neighborhood perceived support**				
**Buckner's Neighborhood Cohesion Instrument**				
Lagged light care	0.028*	0.028*	0.028	0.017
Lagged intensive care	0.025*	0.025*	0.029	0.018
Index of Multiple Deprivation (IMD)	0.159***	0.159***	0.139***	0.176***
**Statistics**				
N	7955	7955	3564	4391

Note: Stars indicate statistical significance *p<0.05, **p<0.01, ***p<0.001. Control variables that account for caregiver characteristics include age, long standing illness and household income.

Supplementary materials

Table 4S. Mediation test using both the Baron and Kenny, and the Zhao, Lynch and Chen approaches.

* Mediation tests from MODEL 6; measure of Overall Social Support (socsup) from family and friends

*** Mediation of Intensive Caregiving in MCS by Overall Social Support ***

Significance testing of indirect effect (unstandardised)

Estimates	Delta	Sobel	Monte Carlo
Indirect effect	-0.010	-0.010	-0.010
Std. Err.	0.022	0.022	0.022
z-value	-0.448	-0.448	-0.442
p-value	0.654	0.654	0.659
Conf. Interval	-0.053 , 0.033	-0.053 , 0.033	-0.056 , 0.034

Baron and Kenny approach to testing mediation
STEP 1 - socsup2:intensive_care1 (X -> M) with B=-0.014 and p=0.653
STEP 2 - mcs2:socsup2 (M -> Y) with B=0.683 and p=0.000
As either STEP 1 or STEP 2 (or both) are not significant,
there is no mediation!

Zhao, Lynch & Chen's approach to testing mediation
STEP 1 - mcs2:intensive_care1 (X -> Y) with B=-1.175 and p=0.014
As the Monte Carlo test above is not significant and STEP 1 is
significant you have direct-only nonmediation (no mediation)!

RIT = (Indirect effect / Total effect)
(0.010 / 1.184) = 0.008
Meaning that about 1 % of the effect of intensive_care1
on mcs2 is mediated by socsup2!

RID = (Indirect effect / Direct effect)
(0.010 / 1.175) = 0.008
That is, the mediated effect is about 0.0 times as
large as the direct effect of intensive_care1 on mcs2!

*** Mediation of Intensive Care in MCS by neighborhood Cohesion (Buckner's Neighborhood Cohesion Instrument) ***

Significance testing of indirect effect (unstandardised)

Estimates	Delta	Sobel	Monte Carlo
Indirect effect	0.072	0.072	0.072
Std. Err.	0.033	0.033	0.034
z-value	2.153	2.153	2.140
p-value	0.031	0.031	0.032
Conf. Interval	0.006 , 0.138	0.006 , 0.138	0.008 , 0.141

Baron and Kenny approach to testing mediation
STEP 1 - nbrsnci3:intensive_care2 (X -> M) with B=0.126 and p=0.026
STEP 2 - mcs3:nbrsnci3 (M -> Y) with B=0.573 and p=0.000
STEP 3 - mcs3:intensive_care2 (X -> Y) with B=-1.175 and p=0.014
As STEP 1, STEP 2 and STEP 3 as well as the Sobel's test above

are significant the mediation is partial!

Zhao, Lynch & Chen's approach to testing mediation
STEP 1 - mcs3:intensive_care2 (X -> Y) with B=-1.175 and p=0.014
As the Monte Carlo test above is significant, STEP 1 is
significant and their coefficients point in opposite
direction, you have competitive mediation (partial mediation)!

RIT = (Indirect effect / Total effect)
(0.072 / 1.103) = 0.065
Meaning that about 7 % of the effect of intensive_care2
on mcs3 is mediated by nbrsnci3!

RID = (Indirect effect / Direct effect)
(0.072 / 1.175) = 0.061
That is, the mediated effect is about 0.1 times as
large as the direct effect of intensive_care2 on mcs3!

*** Mediation of Light Caregiving in MCS by Overall Social Support ***

Significance testing of indirect effect (unstandardised)

Estimates	Delta	Sobel	Monte Carlo
Indirect effect	0.026	0.026	0.026
Std. Err.	0.010	0.010	0.010
z-value	2.747	2.747	2.726
p-value	0.006	0.006	0.006
Conf. Interval	0.008 , 0.045	0.008 , 0.045	0.009 , 0.047

Baron and Kenny approach to testing mediation
STEP 1 - socsup2:light_care1 (X -> M) with B=0.039 and p=0.002
STEP 2 - mcs2:socsup2 (M -> Y) with B=0.683 and p=0.000
STEP 3 - mcs2:light_care1 (X -> Y) with B=-0.165 and p=0.411
As STEP 1, STEP 2 and the Sobel's test above are significant
and STEP 3 is not significant the mediation is complete!

Zhao, Lynch & Chen's approach to testing mediation
STEP 1 - mcs2:light_care1 (X -> Y) with B=-0.165 and p=0.411
As the Monte Carlo test above is significant and STEP 1 is not
significant you have indirect-only mediation (full mediation)!

RIT = (Indirect effect / Total effect)
(0.026 / 0.139) = 0.191
Meaning that about 19 % of the effect of light_care1
on mcs2 is mediated by socsup2!

RID = (Indirect effect / Direct effect)
(0.026 / 0.165) = 0.160
That is, the mediated effect is about 0.2 times as
large as the direct effect of light_care1 on mcs2!

*** Mediation of Light Caregiving in MCS by neighborhood Cohesion (Buckner's Neighborhood Cohesion Instrument) ***

Significance testing of indirect effect (unstandardised)

Estimates	Delta	Sobel	Monte Carlo
Indirect effect	0.029	0.029	0.029
Std. Err.	0.012	0.012	0.012

```
  z-value           |     2.384      |     2.384      |     2.371

  p-value           |     0.017      |     0.017      |     0.018

  Conf. Interval    | 0.005 , 0.054  | 0.005 , 0.054  | 0.006 , 0.055
|--------------------------------------------------------------------------|

  Baron and Kenny approach to testing mediation
  STEP 1 - nbrsnci3:light_care2 (X -> M) with B=0.051 and p=0.013
  STEP 2 - mcs3:nbrsnci3 (M -> Y) with B=0.573 and p=0.000
  STEP 3 - mcs3:light_care2 (X -> Y) with B=-0.165 and p=0.411
           As STEP 1, STEP 2 and the Sobel's test above are significant
           and STEP 3 is not significant the mediation is complete!

  Zhao, Lynch & Chen's approach to testing mediation
  STEP 1 - mcs3:light_care2 (X -> Y) with B=-0.165 and p=0.411
           As the Monte Carlo test above is significant and STEP 1 is not
           significant you have indirect-only mediation (full mediation)!

  RIT  =   (Indirect effect / Total effect)
           (0.029 / 0.136) = 0.216
           Meaning that about 22 % of the effect of light_care2
           on mcs3 is mediated by nbrsnci3!

  RID  =   (Indirect effect / Direct effect)
           (0.029 / 0.165) = 0.178
           That is, the mediated effect is about 0.2 times as
           large as the direct effect of light_care2 on mcs3!
+--------------------------------------------------------------------------+


* Mediation tests from MODEL 7; measure of Emotional Support (support) and Social Strain (strain) from
family and friends


*** Mediation of Intensive Caregiving in MCS by Emotional Support ***

  Significance testing of indirect effect (unstandardised)
+--------------------------------------------------------------------------+
  Estimates         |     Delta      |     Sobel      |  Monte Carlo
|--------------------------------------------------------------------------|
  Indirect effect   |     0.058      |     0.058      |     0.058

  Std. Err.         |     0.044      |     0.044      |     0.044

  z-value           |     1.335      |     1.335      |     1.327

  p-value           |     0.182      |     0.182      |     0.185

  Conf. Interval    | -0.027 , 0.144  | -0.027 , 0.144  | -0.026 , 0.147
|--------------------------------------------------------------------------|

  Baron and Kenny approach to testing mediation
  STEP 1 - support2:intensive_care1 (X -> M) with B=0.059 and p=0.177
  STEP 2 - mcs2:support2 (M -> Y) with B=0.993 and p=0.000
           As either STEP 1 or STEP 2 (or both) are not significant,
           there is no mediation!

  Zhao, Lynch & Chen's approach to testing mediation
  STEP 1 - mcs2:intensive_care1 (X -> Y) with B=-1.156 and p=0.016
           As the Monte Carlo test above is not significant and STEP 1 is
           significant you have direct-only nonmediation (no mediation)!

  RIT  =   (Indirect effect / Total effect)
           (0.058 / 1.097) = 0.053
           Meaning that about  5 % of the effect of intensive_care1
           on mcs2 is mediated by support2!

  RID  =   (Indirect effect / Direct effect)
           (0.058 / 1.156) = 0.051
```

```
          That is, the mediated effect is about 0.1 times as
          large as the direct effect of intensive_care1 on mcs2!
+-----------------------------------------------------------------------------+


*** Mediation of Intensive Caregiving in MCS by Social Strain ***

  Significance testing of indirect effect (unstandardised)
+-----------------------------------------------------------------------------+
  Estimates           |     Delta       |     Sobel       |  Monte Carlo
|-----------------------------------------------------------------------------|
  Indirect effect     |    -0.118       |    -0.118       |    -0.118

  Std. Err.           |     0.051       |     0.051       |     0.052

  z-value             |    -2.308       |    -2.308       |    -2.289

  p-value             |     0.021       |     0.021       |     0.022

  Conf. Interval      | -0.218 , -0.018 | -0.218 , -0.018 | -0.222 , -0.021
|-----------------------------------------------------------------------------|

  Baron and Kenny approach to testing mediation
  STEP 1 - strain2:intensive_care1 (X -> M) with B=0.087 and p=0.017
  STEP 2 - mcs2:strain2 (M -> Y) with B=-1.350 and p=0.000
  STEP 3 - mcs2:intensive_care1 (X -> Y) with B=-1.156 and p=0.016
           As STEP 1, STEP 2 and STEP 3 as well as the Sobel's test above
           are significant the mediation is partial!

  Zhao, Lynch & Chen's approach to testing mediation
  STEP 1 - mcs2:intensive_care1 (X -> Y) with B=-1.156 and p=0.016
           As the Monte Carlo test above is significant, STEP 1 is
           significant and their coefficients point in same direction,
           you have complementary mediation (partial mediation)!

  RIT  =   (Indirect effect / Total effect)
           (0.118 / 1.273) = 0.092
           Meaning that about  9 % of the effect of intensive_care1
           on mcs2 is mediated by strain2!

  RID  =   (Indirect effect / Direct effect)
           (0.118 / 1.156) = 0.102
           That is, the mediated effect is about 0.1 times as
           large as the direct effect of intensive_care1 on mcs2!
+-----------------------------------------------------------------------------+


*** Mediation of Intensive Caregiving in MCS by neighborhood Cohesion (Buckner's Neighborhood Cohesion
Instrument) ***

  Significance testing of indirect effect (unstandardised)
+-----------------------------------------------------------------------------+
  Estimates           |     Delta       |     Sobel       |  Monte Carlo
|-----------------------------------------------------------------------------|
  Indirect effect     |     0.051       |     0.051       |     0.051

  Std. Err.           |     0.025       |     0.025       |     0.025

  z-value             |     2.081       |     2.081       |     2.058

  p-value             |     0.037       |     0.037       |     0.040

  Conf. Interval      | 0.003 , 0.099   | 0.003 , 0.099   | 0.006 , 0.103
|-----------------------------------------------------------------------------|

  Baron and Kenny approach to testing mediation
  STEP 1 - nbrsnci3:intensive_care2 (X -> M) with B=0.126 and p=0.026
  STEP 2 - mcs3:nbrsnci3 (M -> Y) with B=0.407 and p=0.000
  STEP 3 - mcs3:intensive_care2 (X -> Y) with B=-1.156 and p=0.016
           As STEP 1, STEP 2 and STEP 3 as well as the Sobel's test above
           are significant the mediation is partial!
```

```
  Zhao, Lynch & Chen's approach to testing mediation
  STEP 1 - mcs3:intensive_care2 (X -> Y) with B=-1.156 and p=0.016
           As the Monte Carlo test above is significant, STEP 1 is
           significant and their coefficients point in opposite
           direction, you have competitive mediation (partial mediation)!

  RIT  =   (Indirect effect / Total effect)
           (0.051 / 1.104) = 0.046
           Meaning that about  5 % of the effect of intensive_care2
           on mcs3 is mediated by nbrsnci3!

  RID  =   (Indirect effect / Direct effect)
           (0.051 / 1.156) = 0.044
           That is, the mediated effect is about 0.0 times as
           large as the direct effect of intensive_care2 on mcs3!
+-----------------------------------------------------------------------------+


*** Mediation of Light Caregiving in MCS by Emotional Support ***

  Significance testing of indirect effect (unstandardised)
+-----------------------------------------------------------------------------+
  Estimates           |     Delta        |     Sobel        |  Monte Carlo
|-----------------------------------------------------------------------------|
  Indirect effect     |     0.062        |     0.062        |     0.062

  Std. Err.           |     0.018        |     0.018        |     0.018

  z-value             |     3.452        |     3.453        |     3.444

  p-value             |     0.001        |     0.001        |     0.001

  Conf. Interval      | 0.027 , 0.098    | 0.027 , 0.098    | 0.028 , 0.100
|-----------------------------------------------------------------------------|

  Baron and Kenny approach to testing mediation
  STEP 1 - support2:light_care1 (X -> M) with B=0.063 and p=0.000
  STEP 2 - mcs2:support2 (M -> Y) with B=0.993 and p=0.000
  STEP 3 - mcs2:light_care1 (X -> Y) with B=-0.180 and p=0.371
           As STEP 1, STEP 2 and the Sobel's test above are significant
           and STEP 3 is not significant the mediation is complete!

  Zhao, Lynch & Chen's approach to testing mediation
  STEP 1 - mcs2:light_care1 (X -> Y) with B=-0.180 and p=0.371
           As the Monte Carlo test above is significant and STEP 1 is not
           significant you have indirect-only mediation (full mediation)!

  RIT  =   (Indirect effect / Total effect)
           (0.062 / 0.118) = 0.531
           Meaning that about 53 % of the effect of light_care1
           on mcs2 is mediated by support2!

  RID  =   (Indirect effect / Direct effect)
           (0.062 / 0.180) = 0.347
           That is, the mediated effect is about 0.3 times as
           large as the direct effect of light_care1 on mcs2!
+-----------------------------------------------------------------------------+


*** Mediation of Light Caregiving in MCS by Social Strain ***

  Significance testing of indirect effect (unstandardised)
+-----------------------------------------------------------------------------+
  Estimates           |     Delta        |     Sobel        |  Monte Carlo
|-----------------------------------------------------------------------------|
  Indirect effect     |     0.023        |     0.023        |     0.023

  Std. Err.           |     0.019        |     0.019        |     0.019

  z-value             |     1.212        |     1.212        |     1.203
```

```
  p-value           |     0.225      |     0.225      |     0.229

  Conf. Interval    | -0.014 , 0.061  | -0.014 , 0.061  | -0.014 , 0.063
|--------------------------------------------------------------------------|

  Baron and Kenny approach to testing mediation
  STEP 1 - strain2:light_care1 (X -> M) with B=-0.017 and p=0.221
  STEP 2 - mcs2:strain2 (M -> Y) with B=-1.350 and p=0.000
           As either STEP 1 or STEP 2 (or both) are not significant,
           there is no mediation!

  Zhao, Lynch & Chen's approach to testing mediation
  STEP 1 - mcs2:light_care1 (X -> Y) with B=-0.180 and p=0.371
           As the Monte Carlo test above is not significant and STEP 1 is
           not significant you have no effect nonmediation (no mediation)!

  RIT  =   (Indirect effect / Total effect)
           (0.023 / 0.157) = 0.149
           Meaning that about 15 % of the effect of light_care1
           on mcs2 is mediated by strain2!

  RID  =   (Indirect effect / Direct effect)
           (0.023 / 0.180) = 0.130
           That is, the mediated effect is about 0.1 times as
           large as the direct effect of light_care1 on mcs2!
+--------------------------------------------------------------------------+


*** Mediation of Light Caregiving in MCS by neighborhood Cohesion (Buckner's Neighborhood Cohesion
Instrument) ***

  Significance testing of indirect effect (unstandardised)
+--------------------------------------------------------------------------+
  Estimates         |     Delta      |     Sobel      |  Monte Carlo
|--------------------------------------------------------------------------|
  Indirect effect   |     0.021      |     0.021      |     0.021

  Std. Err.         |     0.009      |     0.009      |     0.009

  z-value           |     2.287      |     2.288      |     2.264

  p-value           |     0.022      |     0.022      |     0.024

  Conf. Interval    | 0.003 , 0.039   | 0.003 , 0.039   | 0.004 , 0.040
|--------------------------------------------------------------------------|

  Baron and Kenny approach to testing mediation
  STEP 1 - nbrsnci3:light_care2 (X -> M) with B=0.051 and p=0.013
  STEP 2 - mcs3:nbrsnci3 (M -> Y) with B=0.407 and p=0.000
  STEP 3 - mcs3:light_care2 (X -> Y) with B=-0.180 and p=0.371
           As STEP 1, STEP 2 and the Sobel's test above are significant
           and STEP 3 is not significant the mediation is complete!

  Zhao, Lynch & Chen's approach to testing mediation
  STEP 1 - mcs3:light_care2 (X -> Y) with B=-0.180 and p=0.371
           As the Monte Carlo test above is significant and STEP 1 is not
           significant you have indirect-only mediation (full mediation)!

  RIT  =   (Indirect effect / Total effect)
           (0.021 / 0.159) = 0.131
           Meaning that about 13 % of the effect of light_care2
           on mcs3 is mediated by nbrsnci3!

  RID  =   (Indirect effect / Direct effect)
           (0.021 / 0.180) = 0.116
           That is, the mediated effect is about 0.1 times as
           large as the direct effect of light_care2 on mcs3!
+--------------------------------------------------------------------------+
```

Table 5S. Abbreviated results of selected dynamic panel models including mediation equations using an alternative measure of neighborhood cohesion (Perceived Neighborhood Social Cohesions, PNSC). Standardized adjusted coefficients.

**MCS** Variable	model 1	model 2	model 3	model 4
**Direct effects of caregiving**				
Lagged MCS	0.124***	0.123***	0.128***	0.137***
**Lagged Caregiving**				
(ref. No caregiving)				
Light care	-0.170	-0.171	-0.166	-0.183
Intensive care	-1.132*	-1.135*	-1.148*	-1.134*
Control variables	x	x	x	x
**Direct effects of mediators**				
**Social network perceived support**				
Overall social support			0.551***	
Emotional support				0.895***
Social strain				-1.439***
**Neighborhood perceived support**				
Perceived Neighborhood Social Cohesion	0.059***	0.050***	0.112***	0.067***
Index of Multiple Deprivation (IMD)	-0.033	0.082	-0.020	-0.021
Interaction term with IMD		-0.006		
**Mediators' equations**				
**Indirect effects of caregiving**				
**Social network perceived support**				
**Overall social support**				
Lagged light care			0.039**	
Lagged intensive care			-0.014	
Having a coresident partner			0.037***	
**Emotional support**				
Lagged light care				0.063***
Lagged intensive care				0.059
Having a coresident partner				0.031*
**Social strain**				
Lagged light care				-0.017
Lagged intensive care				0.087*
Having a coresident partner				-0.059***
**Neighborhood perceived support**				
**Perceived Neighborhood Social Cohesion**				
Lagged light care	0.029	0.029	0.029	0.029
Lagged intensive care	-0.145	-0.145	-0.145	-0.145
Index of Multiple Deprivation (IMD)	0.213***	0.213***	0.213***	0.213***

Statistics				
N	7955	7955	7955	7955
Akaike Information Criterion	430000	495000	445000	460000
Bayesian Information Criterion	431000	496000	447000	461000

Note: Stars indicate statistical significance *p<0.05, **p<0.01, ***p<0.001. Control variables that account for caregiver characteristics include age, long standing illness and household income.

Table 6S. Abbreviated results of Model 7 fitted to the General Health Questionnaire (GHQ). Standardized adjusted coefficients.

**GHQ** Variable	**model 7 GHQ**
**Direct effects of caregiving**	
Lagged GHQ	0.105***
**Lagged Caregiving**	
(ref. No caregiving)	
Light care	0.014
Intensive care	0.004
Control variables	x
**Direct effects of mediators**	
**Social network perceived support**	
Emotional support	-0.690***
Social strain	0.072***
**Neighborhood perceived support**	
Buckner's Neighborhood Cohesion Instrument	-0.288***
Index of Multiple Deprivation (IMD)	0.014
**Mediators' equations**	
**Indirect effects of caregiving**	
**Social network perceived support**	
**Emotional support**	
Lagged light care	0.042***
Lagged intensive care	0.015
Having a coresident partner	0.023*
**Social strain**	
Lagged light care	-0.014
Lagged intensive care	0.027*
Having a coresident partner	-0.052***
**Neighborhood perceived support**	
**Buckner's Neighborhood Cohesion Instrument**	
Lagged light care	0.028*
Lagged intensive care	0.025*
Index of Multiple Deprivation (IMD)	0.160***
**Statistics**	
N	7955

Note: Stars indicate statistical significance *p<0.05, **p<0.01, ***p<0.001. Control variables that account for caregiver characteristics include age, long standing illness and household income.

Figure 1S. Path diagrams with the significant associations between caregiving, social support, neighborhood social cohesion, and mental health.

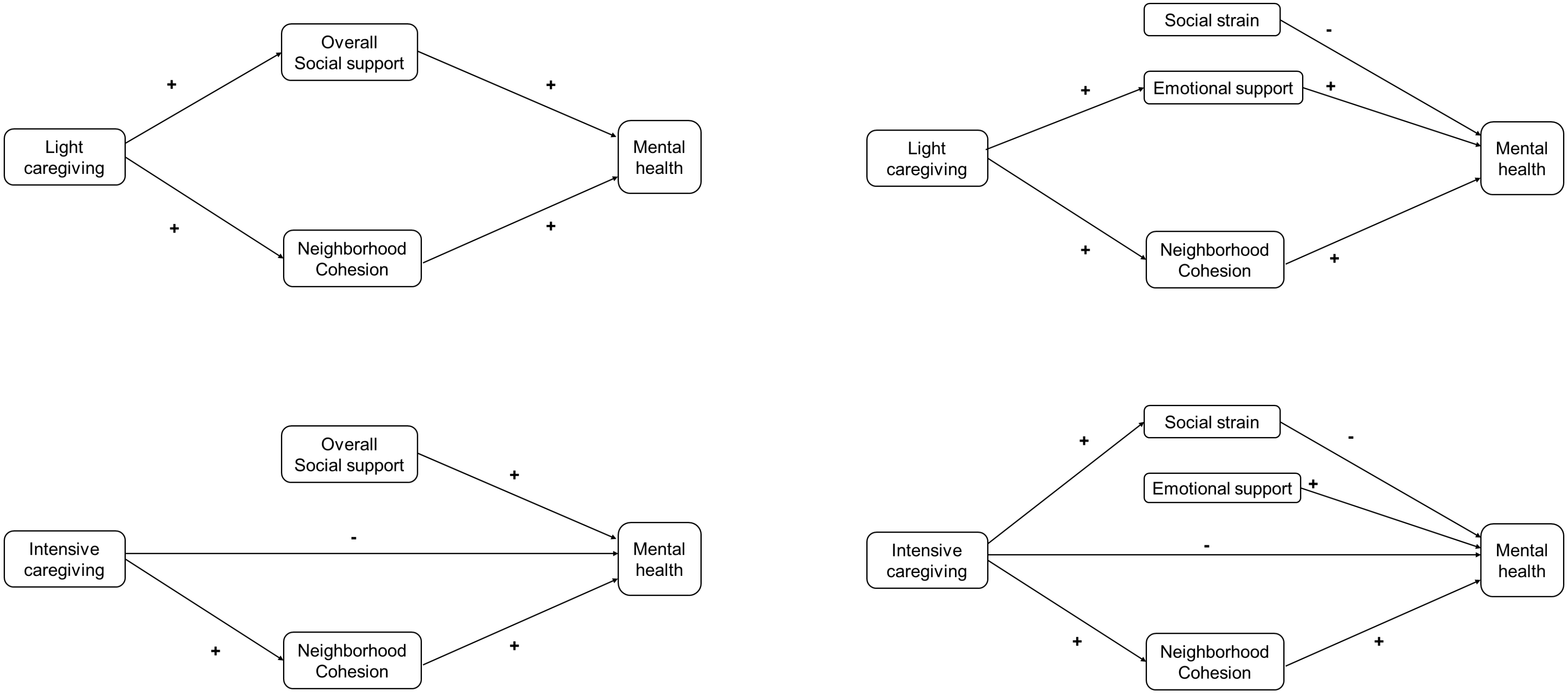

Note: Only significant associations and their direction are shown.
